# Post-Surgical Reassessment of Breast Cancer IHC: Concordance, Δ-Metrics, and Treatment-Relevant Reclassification

**DOI:** 10.3390/diagnostics15243128

**Published:** 2025-12-09

**Authors:** Ramona Andreea Cioroianu, Michael Schenker, Tradian Ciprian Berisha, Virginia-Maria Rădulescu, George Ovidiu Cioroianu, Raluca Chirculescu, Ana Maria Petrescu, Mihaela Popescu, Anda Lorena Dijmărescu, Stelian Ștefăniță Mogoantă

**Affiliations:** 1 Doctoral School, University of Medicine and Pharmacy of Craiova, 200349 Craiova, Romania; 2 Department of Endocrinology, University of Medicine and Pharmacy of Craiova, 200349 Craiova, Romania; 3 Department of Oncology, University of Medicine and Pharmacy of Craiova, 200349 Craiova, Romania; 4 SF Nectarie Oncology Center, 200347 Craiova, Romania; 5 Department of Medical Informatics and Biostatistics, University of Medicine and Pharmacy of Craiova, 200349 Craiova, Romania; 6 Department of Physical Medicine and Rehabilitation, University of Medicine and Pharmacy of Craiova, 200349 Craiova, Romania; 7 Department of Pathology, Filantropia Clinical Hospital, 011132 Bucharest, Romania; 8 Department of Obstetrics-Gynecology, University of Medicine and Pharmacy of Craiova, 200349 Craiova, Romania; 9 Department of Surgery, University of Medicine and Pharmacy of Craiova, 200349 Craiova, Romania

**Keywords:** breast neoplasms, immunohistochemistry, core needle biopsy, surgical specimens, biomarker conversion, neoadjuvant therapy, clinical decision-making, HER2, Ki-67 antigen

## Abstract

**Background/Objectives**: Immunohistochemical (IHC) profiles assessed on core biopsies guide initial therapy in breast cancer; however, paired changes between biopsy and surgical specimens may alter treatment pathways. We aimed to quantify paired biomarker dynamics (ER, PR, HER2, Ki-67) and the proportion of patients undergoing clinically actionable reclassification. **Methods**: We conducted a single-center retrospective study of 79 patients with paired pre- and post-surgical IHC for ER, PR, HER2 (0/1+/2+/3+ with reflex ISH for 2+), and Ki-67 (20% cut-off). Paired categorical shifts were tested with McNemar’s test; agreement was quantified using Cohen’s κ (95% CI); and multivariable logistic regression examined correlates of neoadjuvant chemotherapy (NACT). Two-sided *p* < 0.05 denoted statistical significance. **Results**: Post-surgical reassessment showed measurable conversions: PR-negative increased from 15.19% to 27.85%, while PR-positive decreased 84.81%→72.15%; HER2 3+ contracted 11.39%→6.33% with a parallel rise in 2+ (equivocal) 17.72%→24.05%; Ki-67 < 20% rose 37.97%→56.96%, whereas the >30% category was absent post-surgery. McNemar tests indicated significant paired shifts for PR (*p* = 0.016) and Ki-67 (*p* = 0.009); agreement was substantial for ER (κ = 0.70) and lower for PR (κ = 0.49), HER2 (κ = 0.43), and Ki-67 (κ = 0.29). High proliferation (Ki-67 ≥ 20%) independently predicted NACT (OR = 4.36, 95% CI 1.48–12.80). **Conclusions**: Paired IHC reassessment from biopsy to surgery reveals biomarker conversions that can reclassify therapeutic eligibility (e.g., anti-HER2 candidacy, endocrine strategies). These data support selective confirmation of IHC on resection specimens in routine practice and provide Δ-metrics to inform decision-making; external validation in prospective cohorts is warranted.

## 1. Introduction

Breast cancer represents the most frequent form of cancer diagnosed in women worldwide and is a leading cause responsible for cancer mortality. Globally, in 2020 there were recorded over 2.3 million new cases of breast cancer in females along with approximately 685,000 deaths [1]. Accounting for about 26.9% of all female cancers in Romania, breast cancer has maintained its leading position as the most frequent malignancy in women, while also recording a rise in incidence, which compared to Western Europe is relatively lower but even so it carries a significant mortality rate at a national level [2].

The disease of breast cancer is heterogeneous, consisting of distinct biological sub-types that are characterized by the expression of hormone receptors (ER/PR), the presence of HER2, and the rate of tumor proliferation. These biomarkers not only categorize tumors into subtypes, including Luminal A, Luminal B, HER2-enriched, and triple-negative (TNBC), but also inform therapeutic decisions. For example, endocrine (hormonal) therapy is an option for ER-positive tumors, HER2-overexpressing tumors can be treated with HER2-targeted compounds, and chemotherapy may be recommended for tumors with a high Ki-67 proliferation index. Before treatment is initiated, ER, PR, HER2, and Ki-67 are routinely evaluated through immunohistochemistry on diagnostic core needle biopsies due to their critical roles [3,4,5].

Regarding the prognosis, Luminal A tumors (ER and PR positive, HER2-negative, with low Ki-67) are usually low-grade and present a more favorable prognosis while Luminal B malignancies, which are also hormone receptor-positive but tend to associate a higher grade with a higher Ki-67 index have a somewhat worse prognosis than the Luminal A subtype [6,7,8]. Even though HER2-positive breast cancers are regarded as an aggressive subtype, the development of HER-2 targeted therapies, such as trastuzumab, has greatly improved the outcome of these patients [9]. Contrastingly, TNBC, which are defined by the absence of ER, PR and HER2 receptors, account for about 15–20% of breast cancers and are known for their aggressive behavior and lack of established therapeutic targets. This subtype of tumors also presents higher relapse rates, fueling ongoing research in developing more effective treatments aimed at this specific form of breast cancer [8,10].

Increasingly, patients with more aggressive breast cancers (such as HER2-positive or triple-negative tumors, and some high-grade ER+ tumors) receive neoadjuvant chemotherapy (NACT) prior to surgery. However, subjecting the tumor to therapy before surgical excision poses an essential question: does the tumor’s biomarker profile stay unchanged following treatment? It is established that therapy might cause changes in tumor biology; for example, highly proliferative cells may be selectively destroyed, thereby reducing the Ki-67 index, or resistant clones may develop with altered receptor expression. Even in the absence of medication, studies have noticed some discrepancy between biomarkers assessed on a tiny first biopsy and the ultimate surgical material, due to tumor heterogeneity and sampling variability [3,11,12]. Previous studies on biomarker concordance before and after treatment have yielded inconsistent findings. Numerous studies indicate elevated concordance rates (exceeding 90%) for ER, PR, and HER2 between core biopsy and surgical excision in the absence of interval therapy, implying that receptor status is typically stable without treatment [13,14,15,16]. Others have shown considerable rates of receptor “conversion” when neoadjuvant chemotherapy is administered: for example, loss of ER/PR in a minority of previously positive tumors or occasional gain of HER2 positivity in tumors first considered to be HER2-negative [17,18,19,20]. While all guidelines support confirming receptor status when metastatic relapse occurs (since a tumor’s phenotype can alter over time or under treatment pressure), there is less agreement on routine re-testing of biomarkers after neoadjuvant therapy in early disease [3]. In this context, we conducted a thorough examination of 79 breast cancer patients treated at our center, with a focus on the IHC marker profiles before and after surgery. All patients had invasive breast cancer, with pathology available from biopsy and surgery specimens. We wanted to know how frequently and to what degree HER2, ER, PR, and Ki-67 status changed after surgery (especially in the subset that received neoadjuvant therapy), and whether such changes were linked with patient age, tumor histologic grade, and treatment type. We also looked at the consistency of pre- and post-surgery molecular subtype classification (Luminal A, Luminal B, triple-negative, etc.) and if differences would have affected clinical therapy. By comparing our findings to recent research, we hope to provide light on the reliability of initial biomarker testing and whether a strategy of routine post-surgical biomarker re-evaluation is appropriate in order to maximize tailored treatment.

Against this background, the present study had three main objectives: (i) to quantify the concordance of ER, PR, HER2, and Ki-67 between diagnostic core biopsies and corresponding surgical specimens in a real-world breast cancer cohort; (ii) to measure the frequency and direction of clinically actionable reclassification of these biomarkers; and (iii) to identify treatment contexts—particularly neoadjuvant chemotherapy versus upfront surgery—in which routine post-surgical IHC reassessment is most likely to provide added value for systemic therapy decisions.

## 2. Materials and Methods

### 2.1. Study Design and Patients

The study group included 79 patients diagnosed with invasive breast carcinoma, treated oncologically at the St. Nectarie Oncology Centre, Craiova. Some patients underwent surgery at the County Emergency Clinical Hospital, Craiova, while others were treated surgically in different medical centers. The study was approved by the Ethics Committee of the University of Medicine and Pharmacy of Craiova (Reg. no. 351/17 September 2024). Informed consent was obtained from all subjects involved in the study.

Inclusion criteria were: confirmed histopathological diagnosis, availability of complete clinical and therapeutic data, and the possibility to follow the post-therapeutic evolution. Exclusion criteria were incomplete data or absence of paired immunohistochemistry (IHC) results.

Biological material consisted of breast parenchyma and axillary lymph node specimens. Pre-surgical diagnosis was established by breast core needle biopsy in a subset of patients, while post-surgical samples were collected from excised tumor specimens.

Histopathological examinations were performed either in the Pathology Service of the County Emergency Clinical Hospital, Craiova, or in other accredited pathology laboratories. Clinical data (age at diagnosis, comorbidities, type of surgery, treatments administered) were retrieved from patients’ observation sheets and medical records. Treatment strategies were individualized based on tumor stage, hormone receptor expression, HER2 status, and other molecular features.

### 2.2. Clinical and Pathological Data

Demographic variables (age, sex), comorbidities (hypertension, type II diabetes mellitus, dyslipidemia, heart failure), and clinical presentation (palpable tumor, edema, ulceration) were recorded.

Pathological variables included histologic type, histologic grade (G1–G3), number of examined and invaded lymph nodes, TNM stage, and immunophenotype classification (Luminal A, Luminal B, HER2-enriched, triple-negative).

Histologic type was recorded according to the current WHO classification (invasive ductal carcinoma, invasive lobular carcinoma, and other less frequent subtypes, including mixed ductal–lobular patterns). For the present analysis, invasive ductal and invasive lobular carcinomas were reported separately in subgroup summaries, as their IHC concordance patterns may differ.

### 2.3. Biomarker Assessment

Immunohistochemistry was performed on formalin-fixed, paraffin-embedded tissue slices to assess ER, PR, HER2, and Ki-67 levels. Core biopsy specimens were stained for these markers during the initial diagnosis as part of the usual work-up. Following surgery, the excised tumor specimens underwent repeat IHC testing for the same markers (either on the entire tumor or representative blocks of remaining tumor, as per conventional pathology standards). All IHC testing for this study was performed in the same pathology laboratory using established assays, ensuring methodological consistency for paired comparisons. In accordance with ASCO/CAP criteria, ER and PR status were considered positive if at least 1% of tumor cell nuclei were stained. The percentage of cells staining was recorded, and some cases included Allred scores. The HER2 status was determined using IHC scoring (0 to 3+ scale). Cases with IHC 3+ were classified as HER2-positive; cases with 0/1+ were HER2-negative; and cases with 2+ (equivocal) received reflex testing by fluorescence in situ hybridization (ISH; e.g., FISH or DISH) for HER2 gene amplification to confirm final HER2 status, according to ASCO/CAP recommendations. In a small number of pre-surgery cases, mixed HER2 IHC patterns (1/2+ or 2/3+) were recorded; these were resolved by reflex in situ hybridization, and adjudicated to a final HER2 status according to ASCO/CAP. All analyses used the final categories (0/1+/2+/3+). For treatment-oriented analyses, HER2-positive disease was defined as IHC 3+ or IHC 2+ with ISH-confirmed amplification, whereas IHC 0/1+ and IHC 2+ without amplification were considered HER2-negative.

Ki-67 was calculated as the percentage of tumor cells with nuclear staining (Ki-67 labeling index). For categorical analyses, Ki-67 was classified as low (<20%) or high (≥20%) using a 20% cut-off.

All core biopsies and surgical specimens were processed in the same accredited pathology laboratory, using formalin fixation and paraffin embedding according to routine institutional protocols. IHC was performed on automated platforms with standardized detection systems; ER, PR, and HER2 reporting followed ASCO/CAP recommendations in force at the time of diagnosis, and Ki-67 was classified as low (<20%) or high (≥20%) as described above. Pre- and post-surgical samples were reported within the same pathology service, but in routine practice different staff pathologists may have signed out the cases, and no formal inter-observer reproducibility assessment was undertaken. This potential subjectivity is explicitly acknowledged as a limitation.

### 2.4. Treatment Data

Treatment data included type of surgery (mastectomy, lumpectomy, lymphadenectomy), systemic therapies (neoadjuvant or adjuvant chemotherapy, hormone therapy, targeted molecular therapy), and radiotherapy. Decisions were made in multidisciplinary settings, following national and international guidelines, and adapted to individual tumor biology and clinical context. Within this cohort, 54/79 patients (68.4%) received neoadjuvant chemotherapy before surgery, whereas 25/79 (31.6%) underwent upfront surgery without NACT; subsequent analyses stratify paired IHC changes according to this treatment context.

### 2.5. Statistical Analysis

All clinical, pathological, and therapeutic data were initially collected and organized in Microsoft Excel 2010 spreadsheets. After validation, statistical analyses were performed using IBM SPSS Statistics version 26.0 (IBM Corp., Armonk, NY, USA). Continuous variables were summarized as mean ± SD or median (range); categorical variables as counts and percentages. Group comparisons between independent subgroups used Chi-square tests for categorical variables and Spearman’s correlation for ordinal/continuous variables, as appropriate. Paired changes between pre- and post-surgery ER/PR/HER2/Ki-67 were tested with McNemar’s test; agreement was quantified with Cohen’s κ with 95% confidence intervals. For paired non-parametric continuous comparisons, we used the Wilcoxon signed-rank test. Where applicable, we report 95% CIs for proportions (Wilson) and for paired proportion differences (Newcombe), and we summarize Δ-metrics (post—pre) for interpretability. Clinically actionable reclassification was defined a priori as any paired change altering treatment eligibility (loss/gain of HER2 3+ or ISH-confirmed amplification; PR loss; or a shift across the Ki-67 20% threshold). Multivariable logistic regression identified independent predictors of neoadjuvant chemotherapy, with adjusted odds ratios (ORs) and 95% CIs. Missing data were handled by complete-case analysis; patterns were inspected to support a MCAR/MAR assumption. Model diagnostics included multicollinearity assessment (variance inflation factors) and overall goodness-of-fit. In exploratory analyses, paired comparisons of IHC categories were repeated after stratification by neoadjuvant chemotherapy (NACT vs. upfront surgery) and by histological type (IDC vs. ILC), without formal correction for multiple testing given the limited sample size. Statistical significance was established at *p* < 0.05.

## 3. Results

### 3.1. Baseline Characteristics and Comorbidities

The study included 79 patients with invasive breast carcinoma, with a mean age of 57.1 ± 11.5 years (range: 30–83). Of these, 78 (98.7%) were women and one (1.3%) was a man. Given that male breast carcinoma is managed according to the same IHC-driven treatment algorithms as postmenopausal female disease, we retained this case in the analysis but did not perform sex-stratified subgroup analyses. Hypertension was the most frequent comorbidity (22.8%), followed by dyslipidemia (11.4%), heart failure (11.4%), and type II diabetes mellitus (7.6%). Baseline characteristics are summarized in Table 1. Hypertension prevalence increased significantly with age (*p* = 0.041), while diabetes and dyslipidemia showed no age-related distribution.

### 3.2. IHC Marker Changes Pre-Surgery vs. Post-Surgery (ER, PR, HER2)

Paired analysis of immunohistochemical (IHC) markers before and after surgery summarized within-patient changes in ER, PR, HER2, and Ki-67 (Table 2). Formal inference is reported below using McNemar’s test for categorical shifts, Wilcoxon signed-rank for Ki-67, and Cohen’s κ (95% CIs) for agreement.

#### 3.2.1. Changes in Hormone Receptor Expression (ER and PR)

ER expression remained relatively stable, but a slight increase in ER-negative patients was observed post-surgery, from 10.13% to 13.92%. At the same time, the frequency of ER-positive patients decreased from 89.87% to 86.08%, suggesting possible changes in tumor hormonal status after surgery. Notably, baseline ER positivity was very high in this cohort (71/79, 89.9%), which likely contributed to the strong agreement observed for ER status between biopsy and surgery. Representative immunohistochemical images are presented to illustrate the variability in ER expression observed among patients.

**Figure 1:**
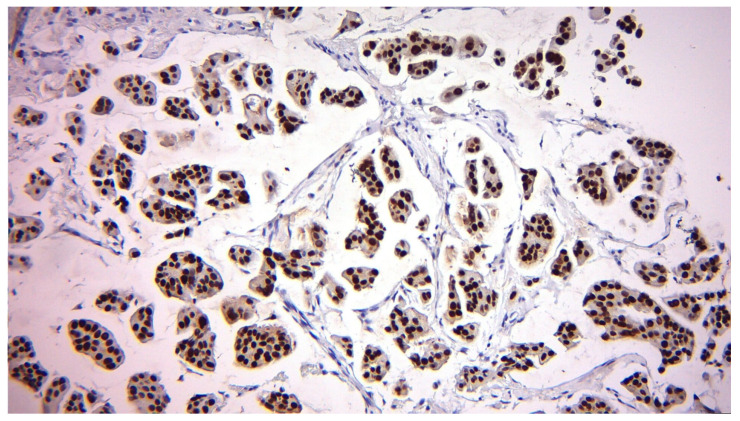
Estrogen receptor (ER) immunohistochemistry showing strong and diffuse nuclear positivity in 98–99% of tumor cells (original magnification 200×). This pattern confirms ER-positive status and supports eligibility for endocrine therapy.

Figure 1 shows a strong and diffuse nuclear staining pattern for ER in approximately 98–99% of tumor cells, indicating high hormone receptor positivity and corresponding to a luminal A phenotype.

**Figure 2:**
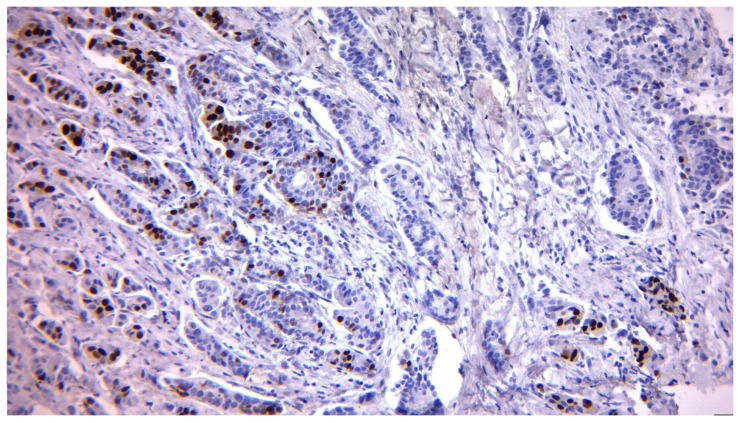
Immunohistochemistry for ER with weak to moderate nuclear staining in ~20–25% of tumor cells, indicative of reduced ER expression (original magnification 200×). Such cases may show partial responsiveness to endocrine therapy.

In contrast, Figure 2 depicts weak to moderate nuclear staining in approximately 20–25% of tumor cells, characteristic of lower ER expression and potentially reduced endocrine therapy responsiveness.

The changes were more pronounced for PR. The frequency of PR-negative patients increased significantly post-surgery, from 15.19% to 27.85%, while the frequency of PR-positive patients decreased from 84.81% to 72.15%. These changes may indicate a loss of PR expression after surgery, which may influence the response to hormonal therapy and the prognosis of patients.

HER2 receptor status showed important variations between pre-surgery and post-surgery. While the percentage of HER2-0 patients remained constant at 55.7%, changes in the distribution of the other categories were observed:

The proportion of HER2 1+ patients increased from 12.66% to 13.92%, indicating possible persistent low expression.

**Figure 3:**
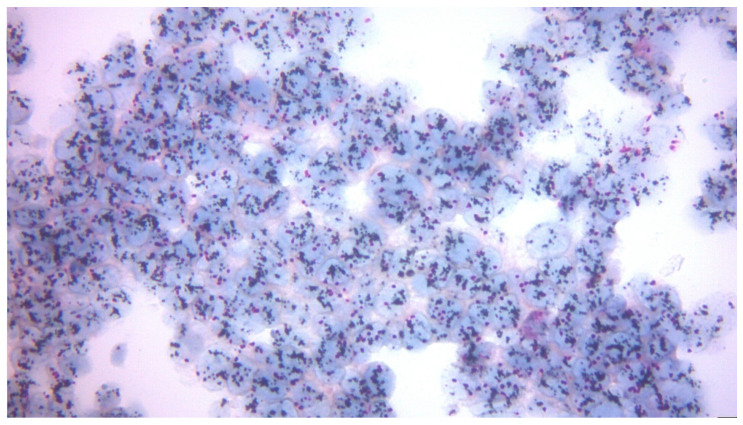
Dual In Situ Hybridization (DISH) for HER2 amplification. The image shows multiple dense clusters of black HER2 gene signals in tumor nuclei, consistent with high-level HER2 gene amplification in a case with equivocal IHC (2+) status (original magnification 400×).

**Figure 4:**
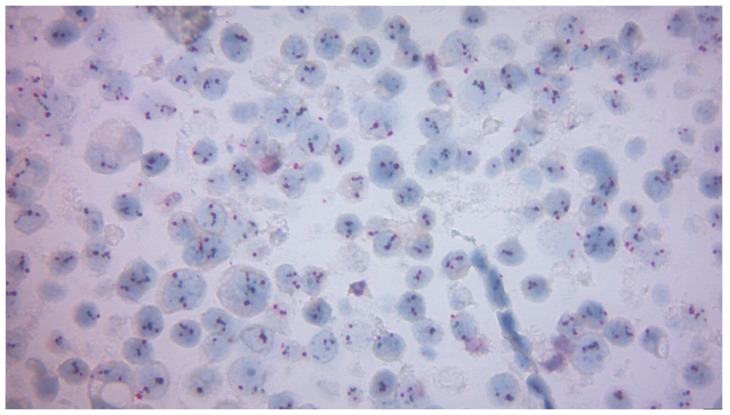
Dual In Situ Hybridization (DISH) showing absence of HER2 gene amplification. The image displays non-clustered HER2 signals dispersed within the tumor nuclei, consistent with HER2-negative status (original magnification 400×).

HER2 2+ (equivocal) expression significantly increased from 17.72% to 24.05%, suggesting a change in tumor heterogeneity post-surgery. In cases with equivocal HER2 (2+) expression by immunohistochemistry, additional testing using in situ hybridization (ISH; e.g., FISH or DISH) was performed to assess HER2 gene amplification status. Figure 3 illustrates a representative example of a high-level HER2 amplification pattern, characterized by clustered black signals within the tumor cell nuclei. This finding confirms gene amplification and supports the classification of the tumor as HER2-positive, making the patient eligible for targeted anti-HER2 therapy, in accordance with ASCO/CAP guidelines. In contrast, Figure 4 presents a case without HER2 gene amplification, confirmed by DISH analysis. The nuclei of tumor cells show scattered, non-clustered signals, with a HER2/CEP17 ratio below the amplification threshold. This pattern supports classification as HER2-negative, in line with ASCO/CAP criteria and corresponding to IHC 0 or 1+ scores.

**Figure 5:**
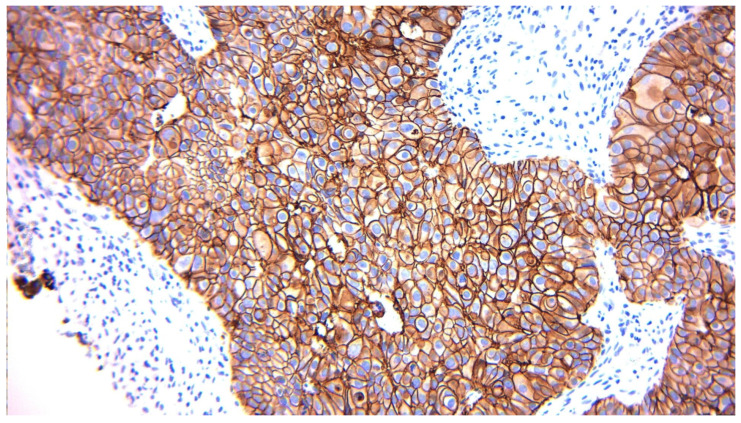
Representative immunohistochemical staining of a breast carcinoma case showing HER2 overexpression (score 3+). Tumor cells exhibit strong, circumferential membranous staining in >10% of cells, consistent with HER2 positivity according to ASCO/CAP guidelines (original magnification 200×).

The HER2 3+ category, corresponding to patients eligible for anti-HER2 therapy, decreased from 11.39% to 6.33%, which may have implications for post-surgery eligibility for molecular-targeted therapies. A representative HER2 3+ tumor specimen is shown in Figure 5, with strong, complete membranous staining of tumor cells, consistent with ASCO/CAP criteria for HER2 positivity and eligibility for targeted anti-HER2 therapy.

The proliferative index Ki-67, a key marker of tumor aggressiveness, also showed marked shifts between pre-surgery and post-surgery measurements. On diagnostic core biopsies, 30/79 (38.0%) tumors had low Ki-67 (<20%) and 49/79 (62.0%) had high Ki-67 (≥20%). In the corresponding surgical specimens, the proportion of low Ki-67 cases increased to 45/79 (57.0%), whereas high Ki-67 decreased to 34/79 (43.0%), indicating a net redistribution from highly proliferative to less proliferative disease (Table 2).

When stratified by neoadjuvant chemotherapy, the global reduction in high Ki-67 was largely driven by the NACT subgroup. Among patients who received NACT, high Ki-67 (≥20%) was present in 40/54 (74.1%) tumors at baseline but decreased to 25/54 (46.3%) after surgery, while low Ki-67 (<20%) increased from 14/54 (25.9%) to 29/54 (53.7%). By contrast, in patients treated with upfront surgery, the proportion of high Ki-67 remained stable at 9/25 (36.0%) both pre- and post-surgery, with balanced bidirectional conversions between low and high categories. These NACT-stratified distributions are summarized in the new Table 3.

Histologically, the cohort comprised 62 (78.5%) invasive ductal carcinomas (IDC), 14 (17.7%) invasive lobular carcinomas (ILC), and 3 (3.8%) other invasive histotypes (ductal in situ or mixed ductal–lobular). Among IDC patients who received neoadjuvant chemotherapy (NACT, *n* = 41), the proportion of high Ki-67 tumors (≥20%) decreased from 33/41 (80.5%) on core biopsies to 20/41 (48.8%) in surgical specimens, while low Ki-67 (<20%) increased from 8/41 (19.5%) to 21/41 (51.2%). In parallel, PR negativity increased from 9/41 (22.0%) to 13/41 (31.7%) after NACT. In ILC patients treated with NACT (*n* = 10), high Ki-67 decreased from 6/10 (60.0%) to 3/10 (30.0%), and low Ki-67 increased from 4/10 (40.0%) to 7/10 (70.0%), whereas PR-negative tumors rose from 1/10 (10.0%) to 2/10 (20.0%). By contrast, in patients managed with upfront surgery, Ki-67 categories remained stable within both IDC (high Ki-67 8/21 [38.1%] before and after surgery) and ILC (high Ki-67 1/4 [25.0%] before and after surgery) subgroups; in this upfront-surgery group, all tumors were PR-positive on core biopsy and PR negativity emerged only postoperatively in 5/21 (23.8%) IDC and 1/4 (25.0%) ILC cases. Given the small numbers, these histotype- and NACT-stratified findings are reported descriptively rather than in a separate table.

This overall distribution is shown in Table 2, while NACT-stratified changes are detailed in the new Table 3.

At a descriptive level, marginal pre- and post-surgical distributions of ER, PR, and HER2 were broadly similar, whereas Ki-67 showed a visible shift from higher to lower categories (Table 2). Analyses aligned with the paired design showed that McNemar’s test identified pre- to post-surgical shifts for PR (*p* = 0.016) and Ki-67 (*p* = 0.009), whereas ER (*p* = 0.371) and HER2 (*p* = 0.453) did not show significant dichotomous change. Concordance estimates complemented these findings: agreement was substantial for ER (κ = 0.70), moderate for PR (κ = 0.49) and HER2 (κ = 0.43), and fair for Ki-67 (κ = 0.29).

#### 3.2.2. Correlation Between Hormonal Markers (ER and PR)

Hormone receptors show significant correlations between pre-surgery and post-surgery values. In particular, ER shows a strong positive correlation between pre-surgery and post-surgery status (Spearman = 0.71), indicating that most patients maintain the same ER status after surgery.

The correlation between pre-surgery and post-surgery expression of the PR receptor is also significantly positive (Spearman = 0.52), suggesting that this marker remains relatively stable after surgery. However, compared to the ER, the correlation is lower, which might indicate a higher susceptibility to post-surgery changes.

#### 3.2.3. Correlation Between Pre-Surgery and Post-Surgery HER2

HER2 receptor expression shows a moderate positive correlation between pre-surgery and post-surgery values (Spearman = 0.41). This correlation is weaker than that of hormone receptors, which might suggest that HER2 status is more prone to change following surgery or adjuvant treatments.

In addition, pre-surgery HER2 was observed to have weak negative correlations with ER and PR, which is consistent with the literature that HER2+ tumors are less frequently ER/PR positive.

The Wilcoxon test was used to evaluate the change in Ki-67. The test result (*p* = 0.005) indicated a statistically significant difference between pre-surgery and post-surgery Ki-67 expression. This may suggest a change in tumor proliferative rate after treatment or surgery, which has important prognostic and therapeutic implications.

#### 3.2.4. Correlation Between Pre-Surgery and Post-Surgery Ki-67

As measured by Ki-67 expression, cell proliferation shows a moderate positive correlation between pre-surgery and post-surgery status (Spearman = 0.31). This suggests that tumors with a high proliferation rate before surgery maintain their characteristics after treatment, but there is also significant variability

#### 3.2.5. Clinically Actionable Reclassification (Pre-Specified)

In line with our pre-specified definition, we next focused on paired biomarker changes with direct therapeutic implications. Overall, 42/79 patients (53.2%) experienced at least one clinically actionable reclassification involving PR, HER2, or Ki-67, although most had only a single marker reclassified.

For hormone receptors, PR was the most labile marker: 12/79 tumors (15.2%) converted from PR-positive to PR-negative between biopsy and surgery, whereas 2/79 (2.5%) converted from PR-negative to PR-positive. Because endocrine therapy decisions in our setting are primarily driven by ER status, isolated PR changes rarely led to complete withdrawal or initiation of endocrine therapy per se; however, PR loss or gain was considered when discussing chemotherapy intensity in borderline luminal B–like presentations.

HER2-positive status (defined as IHC 3+ or IHC 2+ with ISH-confirmed amplification) changed in 9/79 patients (11.4%). Using the final adjudicated HER2 status, 7/79 (8.9%) shifted from HER2-positive to HER2-negative and 2/79 (2.5%) from HER2-negative to HER2-positive between biopsy and surgery. Gains in HER2 positivity may open access to anti-HER2 targeted agents, whereas losses can spare patients unnecessary exposure to such therapies, provided that changes are interpreted in conjunction with histologic and radiologic context.

Ki-67 categories around the 20% cut-off were even more dynamic. A total of 22/79 patients (27.8%) shifted from high (≥20%) to low (<20%) Ki-67, and 7/79 (8.9%) from low to high. As detailed in the NACT-stratified analyses, most high-to-low conversions occurred in patients receiving neoadjuvant chemotherapy, and can support de-escalation of adjuvant chemotherapy when interpreted together with tumor response and intrinsic subtype; conversely, low-to-high shifts argue for cautious escalation of systemic therapy.

A summary of these clinically actionable changes and typical therapeutic implications is presented in Table 4.

### 3.3. IHC Analysis with Immunophenotyping

This analysis evaluated the correlations between immunophenotyping (Luminal A, Luminal B, Triple negative, pre-surgery and post-surgery) and the expression of IHC markers (ER, PR, HER2, and Ki-67). For this purpose, we used Spearman’s coefficient for correlations and the Chi-square test to determine statistically significant differences.

#### 3.3.1. Correlations Between Immunophenotyping and IHC Markers

Pre-surgery, the correlation analysis highlighted a differentiated profile across intrinsic subtypes. Luminal A showed negative correlations with HER2 (Spearman’s ρ = −0.44) and Ki-67 (ρ = −0.67), delineating a phenotype characterized by lower HER2 expression and low proliferation; this configuration is consistent with the literature, which describes Luminal A tumors as poorly proliferative and generally non-HER2+. In contrast, Luminal B correlated negatively with HER2 (ρ = −0.61) and positively with Ki-67 (ρ = 0.56), indicating a higher proliferative rate and a greater frequency of HER2-negative status relative to Luminal A. As expected, the triple-negative subtype exhibited negative correlations with ER (ρ = −0.26) and PR (ρ = −0.32), reflecting the absence of hormone-receptor expression in this biologically aggressive category.

In the post-surgical period, the same trends were maintained, supporting the stability of marker–subtype relationships across the paired assessments: Luminal A remained negatively correlated with HER2 (ρ = −0.56) and Ki-67 (ρ = −0.57), retaining a low-proliferation profile; Luminal B continued to be associated with Ki-67 (ρ = 0.55), indicating increased proliferation even after surgery; and the triple-negative subtype preserved negative correlations with ER (ρ = −0.34) and PR (ρ = −0.68), suggesting the persistence of hormone-receptor negativity at this time point.

To provide an overview of these relationships, Table 5 summarizes the distribution of immunophenotypes by histologic grade. In addition, clinical features such as edema and ulceration were more frequently observed in highly proliferative or triple-negative tumors (*p* < 0.05), supporting the link between aggressive phenotypes and symptomatic presentation. Although some post-surgical categories included a small number of cases, the observed patterns remained in line with expected biological behavior, reinforcing the robustness of these associations.

#### 3.3.2. Significant Differences Between Immunophenotyping and IHC Markers

Using Chi-square (χ^2^) tests of association, we first evaluated pre-surgical relationships between immunophenotype and individual markers. For HER2, significant associations were observed with Luminal A (*p* = 0.004) and with Luminal B (*p* < 0.001), the latter suggesting a more variable distribution of HER2 within this subtype compared with Luminal A. For Ki-67, significant differences across immunophenotypes were likewise evident pre-surgery—Luminal A (*p* < 0.001) and Luminal B (*p* < 0.001)—consistent with lower proliferative activity in Luminal A and higher proliferation in Luminal B and triple-negative tumors. These findings align with the expected biological behavior of intrinsic subtypes and provide a coherent baseline against which paired changes can be interpreted.

In the post-surgical assessment, associations between markers and immunophenotypes were broadly maintained. ER remained associated with Luminal A (*p* = 0.044), indicating preservation of this feature after surgery, while PR showed a marked difference driven by the triple-negative subtype (*p* < 0.001), reflecting the persistent absence of hormone-receptor expression in this group. In addition, HER2 continued to associate with immunophenotype post-surgery—Luminal A (*p* < 0.001) and Luminal B (*p* = 0.002)—suggesting subtype-specific HER2 profiles at both time points. For context, the distribution of immunophenotypes by histologic grade is summarized in Table 3, which complements these χ^2^ results by situating marker–subtype relationships within pathologic severity.

Taken together, the χ^2^ analyses indicate that intrinsic molecular subtypes strongly influence biomarker expression and proliferative profiles both before and after surgery, while the detailed counts and proportions in Table 3 provide a transparent framework for comparing these associations across time points.

### 3.4. Analysis of Relationships and Statistical Differences Between Oncologic Treatments and IHC Markers

This analysis explores correlations and significant differences between the oncologic treatments administered to patients and the expression of immunohistochemical (IHC) markers ER, PR, HER2, and Ki-67, both pre-surgery and post-surgery. Spearman’s correlation coefficient and the Chi-square test were used to identify statistically significant differences.

#### 3.4.1. Correlations Between Treatments and IHC Markers

The correlation analysis revealed the following significant trends. Neoadjuvant chemotherapy shows a negative correlation with PR (Spearman = −0.29) and a positive correlation with Ki-67 (Spearman = 0.36), suggesting that this treatment is more commonly used in patients with tumors with increased proliferation and low PR expression. These observations are consistent with clinical practice, where chemotherapy is more commonly indicated in patients with aggressive tumors.

Adjuvant radiotherapy shows a positive correlation with post-surgery PR (Spearman = 0.26), suggesting that this treatment may be more commonly used in patients who retain post-surgery PR expression.

Adjuvant hormone therapy shows significant correlations with ER and PR. Specifically, a strong positive correlation exists between pre-surgery ER (Spearman = 0.56) and post-surgery ER (Spearman = 0.64), indicating that hormone therapy is used almost exclusively in patients with ER-positive tumors. Also, a positive correlation exists between pre-surgery PR (Spearman = 0.41) and post-surgery PR (Spearman = 0.44), confirming that this treatment is more commonly applied in ER-positive tumors.

These findings emphasize that molecular subtypes not only reflect biomarker profiles but also have significant implications for treatment allocation.

#### 3.4.2. Statistically Significant Differences Between Treatments and IHC Markers

To assess whether treatment allocation differed by IHC markers, we applied Chi-square (χ^2^) tests. For neoadjuvant chemotherapy, significant associations were observed with pre-surgery PR (*p* = 0.026) and pre-surgery Ki-67 (*p* = 0.002), indicating that regimens were more frequently administered to patients with PR-negative tumors and higher proliferation. In addition, adjuvant radiotherapy was associated with post-surgery PR (*p* = 0.041), suggesting that patients who retain PR expression after surgery more often undergo adjuvant irradiation.

Adjuvant hormone therapy showed very strong associations with ER at both time points—pre-surgery ER (*p* < 0.001) and post-surgery ER (*p* < 0.001)—indicating that endocrine therapy is prescribed predominantly (almost exclusively) to ER-positive tumors. Consistently, significant differences were also observed for PR—pre-surgery PR (*p* = 0.001) and post-surgery PR (*p* < 0.001)—confirming preferential use in hormone-receptor–expressing disease. Moreover, HER2 status was associated with endocrine therapy pre-surgery (*p* = 0.031) and post-surgery (*p* = 0.034), suggesting that HER2-negative tumors more frequently receive adjuvant endocrine treatment, in line with molecular classification.

Beyond these associations, the overall distribution of therapeutic strategies is summarized in Table 6. Treatment patterns followed tumor biology: Luminal A cases predominantly received endocrine therapy, whereas triple-negative tumors were more often treated with chemotherapy (*p* < 0.05).

In multivariable logistic regression, high proliferation (Ki-67 ≥ 20%) independently predicted receipt of neoadjuvant chemotherapy (OR = 4.36, 95%CI 1.48–12.80, *p* = 0.007), whereas age, ER, PR, and HER2 were not retained as independent predictors (all *p* > 0.05).

## 4. Discussion

In this cohort study of 79 breast cancer patients, we investigated the stability and changes of key IHC biomarkers (ER, PR, HER2, Ki-67) from the time of initial diagnosis (biopsy) to definitive surgery, in relation to patient age, tumor grade, molecular subtype, and treatment. First, we discovered that the status of hormone receptors (ER and PR) remains generally unaltered after surgery, although HER2 expression and the Ki-67 proliferation index vary more. This finding shows that ER and PR are more stable intra-tumorally, whereas tumor growth and HER2 amplification may be more affected by factors such as tumor heterogeneity or given medications. Zhang et al. found a discordance rate of approximately 9% for ER, 15% for HER2, and 21% for Ki-67 between initial and recurrent tumors [21]. Similarly, in a study conducted by Yousef et al., (2025) with patients treated with neoadjuvant therapy, Ki-67 changed in ~43% of cases post-chemotherapy, significantly more frequently than ER (~12%) [22]. Concordance rates for HER2 and Ki-67 proliferation markers are often lower (≈80–85%), as indicated by You, Kiho et al., (2017), indicating that these markers can alter more frequently due to intra-tumoral heterogeneity or therapy effects [23]. However, several studies have found alterations in PR expression, such as loss of PR in nearly 30% of recurrent tumors in a recent cohort [22]. Such discrepancies suggest that hormone receptor expression may be lost in certain contexts (e.g., under endocrine therapy pressure), but overall, more than 85% of tumors maintain their initial hormonal status, indicating that instituted endocrine treatment will remain effective in the long run [21]. In line with our statistical analysis, ER status showed substantial stability pre- and post-surgery (κ = 0.70, McNemar *p* = 0.371), whereas PR and Ki-67 exhibited significant shifts and lower concordance (κ = 0.49 and 0.29; McNemar *p* = 0.016 and *p* = 0.009, respectively). This variability underscores the need for careful reassessment of these biomarkers when planning adjuvant treatment strategies.

Second, we found a strong association between intrinsic breast cancer subtypes and IHC marker profiles. The distribution of molecular subtypes (Luminal A, Luminal B, HER2-enriched, triple-negative) in the cohort was consistent with the expected patterns based on ER, PR, HER2, and Ki-67 expression. These subtype-marker associations shown in our investigation are entirely compatible with current literature, which emphasizes that IHC profiles dictate intrinsic categorization and, implicitly, prognosis. As a prognostic marker, Ki-67 was used to differentiate luminal A and luminal B breast cancer molecular subtypes with a cutoff of 14%, which was changed to >20% in 2013 by the breast expert at the St Gallen International Breast Cancer Conference [24]. This molecular classification has significant clinical implications: Luminal A cancers, which are often low-grade and slow-growing, have the greatest prognosis, whereas Triple-negative and some HER2-enriched tumors exhibit more aggressive evolution and a poorer prognosis in the absence of targeted therapy [25]. Thus, accurate subtype identification using IHC markers remains critical for predicting disease progression and guiding optimal therapy.

Our analysis indicates that the treatment strategies were closely associated with the IHC-defined subtype and marker profile of each tumor. Patients with more aggressive tumor biology, such as those with PR-negative status or high Ki-67, were significantly more likely to have received neoadjuvant chemotherapy. In terms of clinical practice, this approach is consistent with the most recent patterns in oncology: neoadjuvant chemotherapy is frequently employed to downstage the disease in hormone receptor–negative and extremely proliferative tumors, which are more chemosensitive [26]. For example, triple-negative tumors (usually PR-negative, high Ki-67) frequently received preoperative treatment in our sample, which is predicted given that TNBCs often react well to chemotherapy, achieving pathologic full remission in approximately 40% of cases, as also reported by Srivastava et al. in their study on triple-negative low Ki-67 breast cancers (2022) [27]. Our multivariable analysis further confirmed that high Ki-67 (≥20%) was the only independent predictor of neoadjuvant chemotherapy (OR = 4.36, 95% CI 1.48–12.80, *p* = 0.007), reinforcing the central role of proliferation index in guiding systemic treatment decisions.

We also found patterns in adjuvant therapy that corresponded to receptor profiles. In our series, adjuvant radiation was more commonly used for PR-positive (luminal) cancers. This is most likely due to surgical options: luminal tumors are frequently early-stage and can be treated with breast-conserving surgery followed by radiotherapy, according to a meta-analysis by Pan et al. [28]. Finally, our cohort’s hormonal (endocrine) medication use was correctly guided by ER/PR status. Almost all patients with ER and/or PR positive tumors received adjuvant endocrine therapy, in accordance with worldwide guidelines that require at least 5 years of endocrine treatment for hormone receptor-positive breast cancer [29]. Overall, our findings confirm that tumor biology—particularly proliferation index and hormone receptor status—was the main determinant of therapeutic decisions, underscoring the importance of reassessing biomarkers after surgery and reinforcing the paradigm of individualized, biology-driven treatment in breast cancer.

Among 79 paired cases, several conversions have direct therapeutic implications. First, the HER2 3+ proportion decreased 11.39%→6.33%, potentially reducing eligibility for anti-HER2 regimens in a subset of patients, while HER2 2+ increased, prompting reflex testing to clarify status. Second, PR loss was common (PR-negative 15.19%→27.85%), supporting re-evaluation of endocrine therapy plans when surgery is performed after diagnosis. Third, a shift toward lower proliferation was observed (Ki-67 < 20% 37.97%→56.96%, disappearance of Ki-67 > 30%), consistent with de-escalation signals in some cases. Together, these paired changes indicate that post-surgical IHC can reclassify treatment pathways; we therefore provide Δ-metrics and agreement indices (McNemar, κ with 95% CI) to support selective re-testing in routine practice.

Our findings complement recent prospective data on IHC dynamics under neoadjuvant chemotherapy. Pandya et al. reported significant post-NACT changes in ER, PR, HER2, and a marked reduction in Ki-67 in a cohort of 62 women, with nearly one fifth of patients acquiring positivity for at least one receptor after treatment [30]. In our real-world series, the largest shifts in PR and Ki-67 likewise clustered in the NACT subgroup, whereas patients treated with upfront surgery showed predominantly stable IHC profiles. However, as highlighted by Pandya et al. and others, a decrease in Ki-67 does not uniformly translate into improved long-term outcomes, because residual resistant clones and spatial heterogeneity may still drive relapse [30]. In our cohort, the lack of systematic follow-up precludes direct analysis of recurrence or survival, so these biomarker shifts should be interpreted in conjunction with tumor response, residual disease burden and intrinsic subtype when individualizing post-surgical treatment.

This study has several important limitations. First, breast cancer is intrinsically heterogeneous at the spatial and clonal level, and discordance between core biopsy and surgical specimens is an expected consequence of sampling different tumor regions rather than a novel biological observation. Our contribution is therefore pragmatic rather than mechanistic, quantifying how often pre-surgical IHC categories would, in this setting, lead to reclassification and potential treatment change. Second, the cohort is relatively small (*n* = 79) for a common disease entity, which limits the precision of subgroup estimates, particularly for lobular carcinomas and for the male patient, and precludes robust multivariable modelling of outcomes. Third, pre- and post-surgical IHC assessments were performed within the same pathology service but without formal inter-observer reproducibility studies; subjective variability in scoring may therefore contribute to some of the observed discrepancies. Finally, the retrospective, single-center design and the absence of long-term outcome data mean that we cannot directly quantify how biomarker shifts translated into recurrence or survival differences. These caveats argue for cautious extrapolation and for prospective validation in larger, multi-center cohorts.

## 5. Conclusions

In this single-center, retrospective cohort of 79 patients with invasive breast carcinoma, paired immunohistochemical assessment of ER, PR, HER2, and Ki-67 on diagnostic core biopsies and surgical specimens confirmed a high overall level of concordance, but also documented a non-negligible subset of clinically relevant reclassifications. Most categorical shifts in proliferative index and hormone receptor status clustered in patients receiving neoadjuvant chemotherapy, whereas biomarker profiles remained largely stable in those treated with upfront surgery.

Taken together, these findings refine rather than overturn current practice: they support the use of pre-operative IHC as a generally robust basis for systemic therapy selection, while highlighting concrete scenarios in which targeted post-surgical re-evaluation is most likely to be informative—particularly after neoadjuvant chemotherapy and when clinical, radiologic, and pathological features are discordant. By quantifying the frequency, direction, and treatment implications of IHC reclassification in a real-world, biology-driven pathway, our study offers pragmatic data that can help clinicians balance the added value of repeat IHC against additional workload and costs. Although the modest sample size, single-center design, lack of formal inter-observer reproducibility assessment, and absence of systematic outcome data warrant cautious interpretation of effect sizes, the consistency of patterns across treatment and histologic subgroups supports the clinical plausibility of our observations. Future larger, prospective, multi-center studies with standardized pathology workflows, independent double reading, and long-term follow-up will be important to validate and extend these results and to clarify how IHC reclassification ultimately translates into differences in patient outcomes.

## Figures and Tables

**Table 1:** Baseline demographic and clinical characteristics of the study population.

Variable	*n* (%) or Mean ± SD
Total patients	79
Age (years)	57.1 ± 11.5 (range: 30–83)
Gender	Female: 78 (98.7%), Male: 1 (1.3%)
Hypertension (HTA)	18 (22.8%)
Type II Diabetes Mellitus	6 (7.6%)
Dyslipidemia	9 (11.4%)
Heart failure	9 (11.4%)

Values are presented as absolute numbers and percentages, unless otherwise specified. SD = standard deviation; HTA = hypertension.

**Table 2:** Distribution of IHC marker categories pre- and post-surgery (*n* = 79).

Marker	Category	Pre-Surgery, *n* (%)	Post-Surgery, *n* (%)
ER	Negative	8 (10.13)	11 (13.92)
	Positive	71 (89.87)	68 (86.08)
PR	Negative	12 (15.19)	22 (27.85)
	Positive	67 (84.81)	57 (72.15)
HER2	0	44 (55.7)	44 (55.7)
	1+	10 (12.66)	11 (13.92)
	2+	14 (17.72)	19 (24.05)
	3+	9 (11.39)	5 (6.33)
Ki-67	<20%	30 (37.97)	45 (56.96)
	20–30%	49 (62.03)	34 (43.04)
	>30%	-	-

Values are *n* (%); percentages calculated from the total cohort (*n* = 79). “-” = not observed.

**Table 3:** Distribution of IHC marker categories before and after surgery, stratified by neoadjuvant chemotherapy.

Marker	Category	Pre-Surgery, NACT (*n* = 54) *n* (%)	Post-Surgery, NACT (*n* = 54) *n* (%)	Pre-Surgery, Upfront Surgery (*n* = 25) *n* (%)	Post-Surgery, Upfront Surgery (*n* = 25) *n* (%)
ER	Negative	7 (12.96)	8 (14.81)	1 (4.00)	3 (12.00)
	Positive	47 (87.04)	46 (85.19)	24 (96.00)	22 (88.00)
PR	Negative	12 (22.22)	16 (29.63)	0 (0.00)	6 (24.00)
	Positive	42 (77.78)	38 (70.37)	25 (100.00)	19 (76.00)
HER2	0	29 (53.70)	25 (46.30)	15 (60.00)	19 (76.00)
	1+	6 (11.11)	9 (16.67)	4 (16.00)	2 (8.00)
	2+	9 (16.67)	15 (27.78)	5 (20.00)	4 (16.00)
	3+	8 (14.81)	5 (9.26)	1 (4.00)	0 (0.00)
Ki-67	<20%	14 (25.93)	29 (53.70)	16 (64.00)	16 (64.00)
	≥20%	40 (74.07)	25 (46.30)	9 (36.00)	9 (36.00)

Values are *n* (%). Percentages are calculated within each treatment subgroup (NACT vs. upfront surgery). NACT: neoadjuvant chemotherapy.

**Table 4:** Clinically actionable biomarker reclassification and potential therapeutic implications (*n* = 79).

Biomarker	Clinically Actionable Change (Pre → Post)	Patients *n* (%)	Typical Therapeutic Implication
PR	PR-positive → PR-negative	12 (15.2)	Less favorable endocrine profile; may support chemotherapy escalation
	PR-negative → PR-positive	2 (2.5)	Endocrine therapy becomes more clearly indicated
HER2	HER2-positive → HER2-negative	7 (8.9)	Potential de-escalation or discontinuation of anti-HER2 targeted therapy
	HER2-negative → HER2-positive	2 (2.5)	New eligibility for adding anti-HER2 targeted therapy
KI-67	High (≥20%) → Low (<20%)	22 (27.8)	Supports de-escalation of chemotherapy in responding, high-proliferative BC
	Low (<20%) → High (≥20%)	7 (8.9)	Favors intensification of systemic therapy
Any marker	≥1 clinically actionable change in PR, HER2, or Ki-67 (above)	42 (53.2)	Treatment plan revisited in multidisciplinary discussion

**Table 5:** Distribution of immunophenotypes by histologic grade.

Histologic Grade		Luminal A	Luminal B	Triple-Negative
Pre-surgery	G1	4	7	0
	G2	14	9	1
	G3	5	0	2
Post-surgery	G1	6	0	0
	G2	4	3	3
	G3	1	1	1

Luminal A: ER+ and/or PR+, HER2−, Ki-67 < 20%; Luminal B: ER+ and/or PR+, HER2−/+, Ki-67 ≥ 20%; Triple-negative: ER−, PR−, HER2−. Grading according to histologic evaluation of pre- and post-surgical specimens.

**Table 6:** Therapeutic strategies applied in the study population.

Therapy	*n* (%)
Mastectomy	60 (75.9%)
Lumpectomy	19 (24.1%)
Lymphadenectomy	68 (86.1%)
Neoadjuvant chemotherapy	54 (68.4%)
Adjuvant chemotherapy	21 (26.6%)
Adjuvant radiotherapy	45 (57.0%)
Neoadjuvant hormone therapy	7 (8.9%)
Adjuvant hormone therapy	67 (84.8%)
Targeted molecular therapy	12 (15.2%)

Values represent the number and percentage of patients receiving each treatment modality. Percentages calculated from the total cohort (*n* = 79).

## Data Availability

The raw data supporting the conclusions of this article will be made available by the authors on request.

## Abbreviations

The following abbreviations are used in this manuscript:

HER2	Human Epidermal growth factor Receptor 2
CEP17	Chromosome Enumeration Probe 17
ISH	In Situ Hybridization
FISH	Fluorescence In Situ Hybridization
ASCO/CAP	American Society of Clinical Oncology/College of American Pathologists
PR	Progesterone Receptor
ER	Estrogen Receptor
IHC	Immunohistochemistry
TNBC	Triple-Negative Breast Cancer
NACT	Neoadjuvant Chemotherapy
OR	Odds Ratio
CI	Confidence Interval
SD	Standard Deviation
